# Supplementary material for: Saccharomyces boulardii (CNCM I-745) improves intestinal damage in sepsis by remodeling intestinal flora structure
Source: Front Cell Infect Microbiol. 2025 Jul 31;15:1602792. doi: 10.3389/fcimb.2025.1602792 (PMC12350329; doi:10.3389/fcimb.2025.1602792)
Supplement: Supplementary file 1 [file DataSheet1.zip › summary/summary/6_Taxonomy_community/1_Taxonomy_profiling/6_Genus/Group/Genus_abund_top30.Group.cluster.pdf]

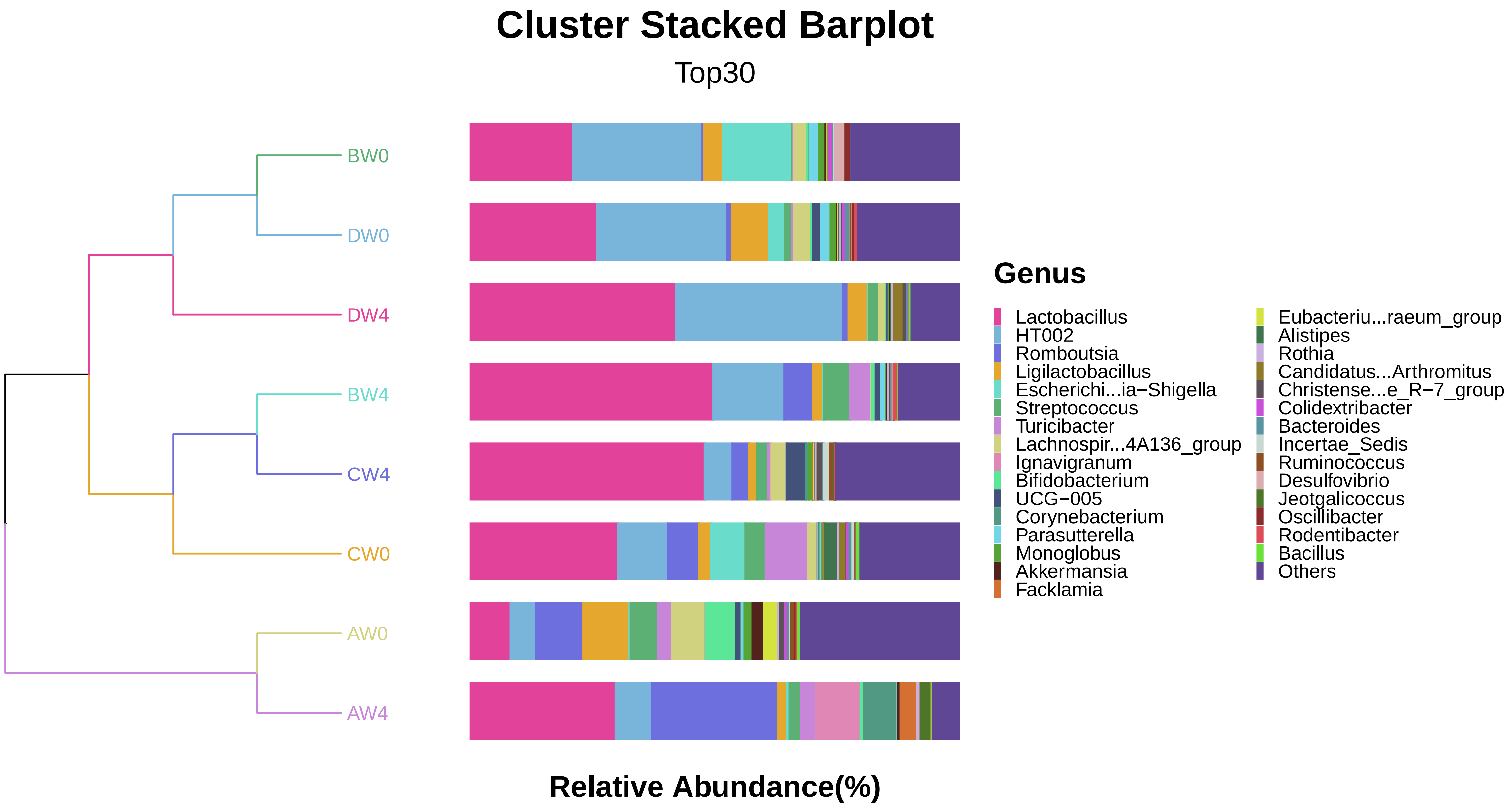

Cluster Stacked Barplot
Top30
BW0
DW0
DW4
BW4
CW4
CW0
AW0
AW4
Relative Abundance(%)
Genus
Lactobacillus
HT002
Romboutsia
Ligilactobacillus
Escherichi...ia−Shigella
Streptococcus
Turicibacter
Lachnospir...4A136_group
Ignavigranum
Bifidobacterium
UCG−005
Corynebacterium
Parasutterella
Monoglobus
Akkermansia
Facklamia
Eubacteriu...raeum_group
Alistipes
Rothia
Candidatus...Arthromitus
Christense...e_R−7_group
Colidextribacter
Bacteroides
Incertae_Sedis
Ruminococcus
Desulfovibrio
Jeotgalicoccus
Oscillibacter
Rodentibacter
Bacillus
Others
